# Deep learning to promote health through sports and physical training

**DOI:** 10.3389/fpubh.2025.1583581

**Published:** 2025-05-27

**Authors:** Xinyue Li

**Affiliations:** Department of Sports, Nanjing Forestry University, Nanjing, China

**Keywords:** deep learning, health improvement, sports science, physical training, time-series analysis, artificial intelligence

## Abstract

**Background:**

Physical activity plays a crucial role in maintaining health and preventing chronic diseases. However, accurately assessing the impact of sports and physical training on health improvement remains a challenge. Recent advancements in deep learning and time-series analysis offer an opportunity to develop more personalized and accurate predictive models for assessing health improvement trends.

**Methods:**

This study proposes a Health Improvement Score (HIS) prediction model based on a sequence-to-sequence deep learning architecture with Long Short-Term Memory (LSTM) networks and an attention mechanism. The model integrates heterogeneous time-series data, including physiological parameters (heart rate, blood oxygen levels, respiration rate), activity metrics (steps, distance, calories burned), sleep patterns, and body measurements. A dataset comprising 384 participants over a 32-day period was used to train and evaluate the model.

**Results:**

The experimental results demonstrate that the proposed HIS prediction model outperforms traditional and machine learning-based models. It achieves 22.8% lower Mean Absolute Error (MAE), 19.3% lower Root Mean Squared Error (RMSE), 6.5% higher *R*^2^, and 7.9% higher Explained Variance Score (EVS) compared to competitive models.

**Conclusion:**

The proposed HIS prediction model effectively captures complex temporal dependencies and improves the accuracy of health improvement predictions.

## 1 Introduction

Deep learning and artificial intelligence (AI) have become powerful tools in public health and sports science. The availability of wearable devices, mobile health applications, and large-scale datasets has allowed AI-driven models to analyze physiological metrics, activity patterns, and training behaviors. These insights help in designing personalized fitness programs, optimizing training efficiency, and improving public health outcomes (1). AI has been successfully used for evaluating physical training quality and predicting individual performance in various sports-related tasks (2).

The integration of AI into physical education has also been explored in academic and professional settings. For instance, studies on Denmark's sports education strategy emphasize the need for pedagogically competent physical training instructors who incorporate motion-focused technologies in their teaching (3). Similarly, research on university students in China found that while many students express interest in sports, factors like academic stress and lack of resources prevent them from participating regularly (4). AI-driven personalized interventions can help address these barriers by making training more accessible and tailored to individual needs.

### 1.1 Background

Physical activity is essential for maintaining overall health. However, there is a growing concern about declining fitness levels among children, students, and the general population. A study conducted in Japan analyzed 34 years of physical fitness data from school students and found significant variations in performance based on training methods and societal influences (5). Similarly, research on children and young people in Ukraine indicated a decline in the number of healthy students over time, emphasizing the need for better sports and fitness programs in schools (6).

Traditional methods of physical education have often been rigid and fail to consider individual differences in performance and motivation. A study on college students' engagement in physical education classes found that fostering sports interest plays a crucial role in sustaining long-term participation (7). AI-driven approaches can help by personalizing training routines based on individual fitness levels, psychological factors, and real-time performance tracking (8).

The importance of technology in training is evident in various domains. Research on firefighters showed that physical health parameters such as VO2 max and muscular strength are strong predictors of job-specific performance (2). Similarly, exergames, or interactive fitness games, have been developed to maintain heart rate levels and optimize training loads through AI-based adaptive control (9). These examples highlight how AI can be used to create tailored training regimens that improve physical performance and health outcomes.

### 1.2 Related work

Many researchers have explored the application of deep learning in evaluating and improving physical training. AI models have been successfully used to assess public sports training quality and optimize performance (1). The use of AI in sports training is not limited to performance enhancement; it also extends to health monitoring and injury prevention. For example, studies have demonstrated that AI-driven models can predict injury risks by analyzing movement patterns and biomechanical data (10).

Several studies have focused on the psychological and motivational aspects of physical training. A study on Moroccan high school students found that commitment is a crucial mental skill that influences performance in physical education and sports (8). This finding suggests that AI-based training programs should incorporate motivational strategies to enhance adherence and engagement. Another study emphasized the role of mental resilience in sports training, showing that psychological preparedness significantly affects physical performance outcomes (11).

In addition to structured sports programs, AI has been applied in specialized physical activities. For example, dance training has been shown to improve motor functions and well-being in patients with Parkinson's disease (12). Similarly, studies on Pilates and Bodyflex techniques have demonstrated their effectiveness in enhancing psychophysiological capabilities among students (13). AI can further optimize these training methodologies by analyzing movement efficiency and recommending personalized exercise modifications.

Educational environments also play a critical role in shaping physical health habits. Research on school health approaches suggests that integrating structured fitness programs into the curriculum can improve students' overall well-being (14). Similarly, the use of health-saving technologies in teacher training programs has been identified as an effective way to promote physical activity among younger generations (15).

Although classical time-series methods such as ARIMA (16) and SARIMA (17) excel at modeling single-variable trends, they cannot fuse multiple feature streams. Support-Vector Regression (SVR) (18) and hybrid ARIMA-SVR (19) introduce nonlinearity but still treat physiological, activity, and sleep series independently. Likewise, deep-learning approaches–including plain LSTM (20), GRU (21), and CNN-LSTM (22) architectures–capture sequential dependencies but rarely integrate heterogeneous inputs or dynamically highlight the most informative time steps. Consequently, none of these prior models fully exploits the rich, multi-domain data now available from wearable platforms, motivating our multi-domain, attention-based sequence-to-sequence framework.

### 1.3 Gaps in literature

Existing approaches to health-forecasting often excel within a single data modality but falter when faced with today's rich, multi-domain streams. Classical statistical models, ARIMA (16) and SARIMA (17), capture univariate trends effectively but cannot fuse heterogeneous inputs such as physiological, activity, and sleep metrics. Hybrid methods like ARIMA-SVR (19) introduce nonlinear fitting for individual series, yet still treat each feature domain in isolation.

Deep-learning architectures (plain LSTM (20), GRU (21), and CNN-LSTM (22)) advance beyond manual feature engineering by learning temporal dependencies, but they typically perform only early- or late-fusion via feature concatenation. This simplistic merging ignores domain-specific noise characteristics and fails to differentiate critical events–e.g. acute heart-rate spikes during intense exercise or phases of fragmented sleep–which may carry outsized importance for health predictions.

Attention mechanisms have revolutionized sequence modeling in NLP and vision (23), enabling dynamic weighting of input elements based on learned relevance. However, their application to multi-modal health time series remains scarce. Few works adapt attention to align and prioritize disparate physiological and behavioral streams, and none provide an end-to-end framework that both synchronizes multi-domain inputs and generates interpretable attention profiles in the context of personalized health forecasting.

Taken together, these gaps underscore the need for a unified, attention-driven sequence-to-sequence model that can (1) seamlessly fuse diverse time-series domains, (2) automatically highlight the most predictive temporal events, and (3) offer transparency into which days and metrics drive each individual's HIS predictions.

### 1.4 Reserach motivation

Despite growing interest in AI-driven health forecasting, existing approaches such as ARIMA (16), SVR (18), and even standard LSTM models (20) generally treat each data modality in isolation, focusing only on physiological signals, activity counts, or sleep metrics. In addition, these methods apply a uniform temporal weighting in all time steps, making them ill suited to detect and leverage anomalous or particularly informative intervals (for example, an acute heart rate spike or a night of severely disrupted sleep). As a result, they do not fully exploit the heterogeneous streams of data now available from modern mobile and wearable health platforms and offer limited interpretability for personalized intervention design.

In contrast, our proposed Health Improvement Score (HIS) predictor combines multidomain time series: heart rate, blood oxygen levels, step counts, sleep phase distributions, and periodic body measurements into a single sequence-to-sequence LSTM architecture. By embedding an attention mechanism between the recurrent layers and the final output, the model learns to dynamically assign higher weights to the most predictive time steps and feature channels.

### 1.5 Contributions

The proposed HIS prediction model introduces several novel elements to improve health assessment through deep learning. The key contributions of this paper are as follows.

1. A novel sequence-to-sequence model is designed to capture both short-term and long-term temporal dependencies in health-related data.
2. An integrated attention mechanism is also used to dynamically weighs different time steps. This allows the proposed model to focus on the most relevant physiological and activity patterns.
3. The proposed model effectively combines heterogeneous data sources, such as physiological, activity, sleep, and body metrics.
4. To ensure personalized fitness plans and sustainable health benefits, AI-based recommendations are provided to optimize sports and physical training.

## 2 Proposed Health Improvement Score (HIS) prediction model

### 2.1 Dataset

**Figure 1:**
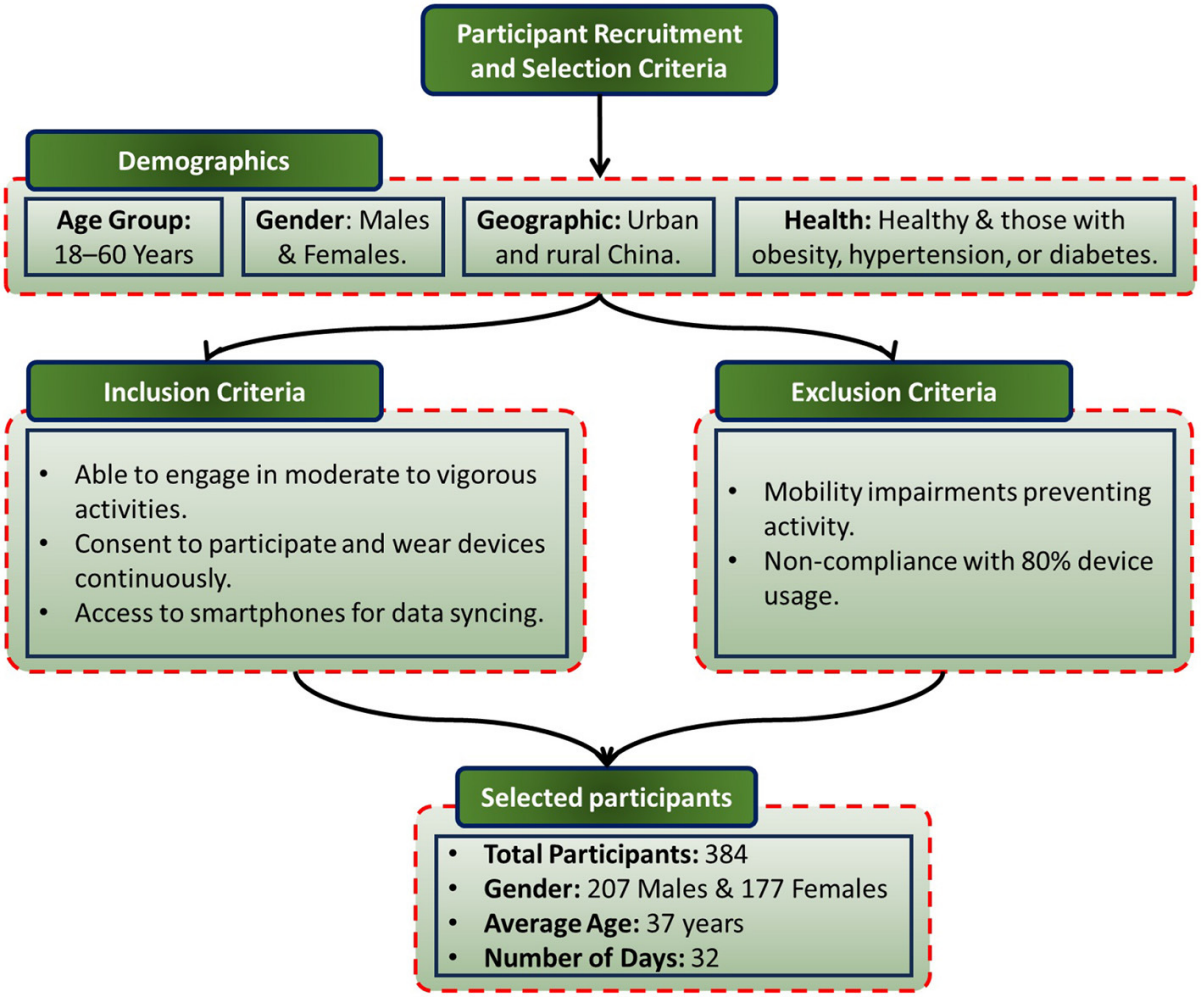
Flowchart illustrating the recruitment and selection process for study participants, including inclusion and exclusion criteria.

The dataset for this research comprises comprehensive physiological, activity, sleep, and body metrics collected from 384 participants over a period of 32 days. Participants were selected following a rigorous recruitment process detailed in Figure 1, which outlines the Participant Recruitment and Selection Criteria. The demographic distribution includes 207 males and 177 females, with an average age of 37 years. This diverse sample ensures a robust representation for the study's objectives. Participants with pre-existing cardiovascular, respiratory, or metabolic disorders were excluded to avoid confounding effects on physiological measurements. Any individual whose record contained more than 20% missing values across the 32-day period was also removed. The remaining gaps were imputed via forward-fill.

**Figure 2:**
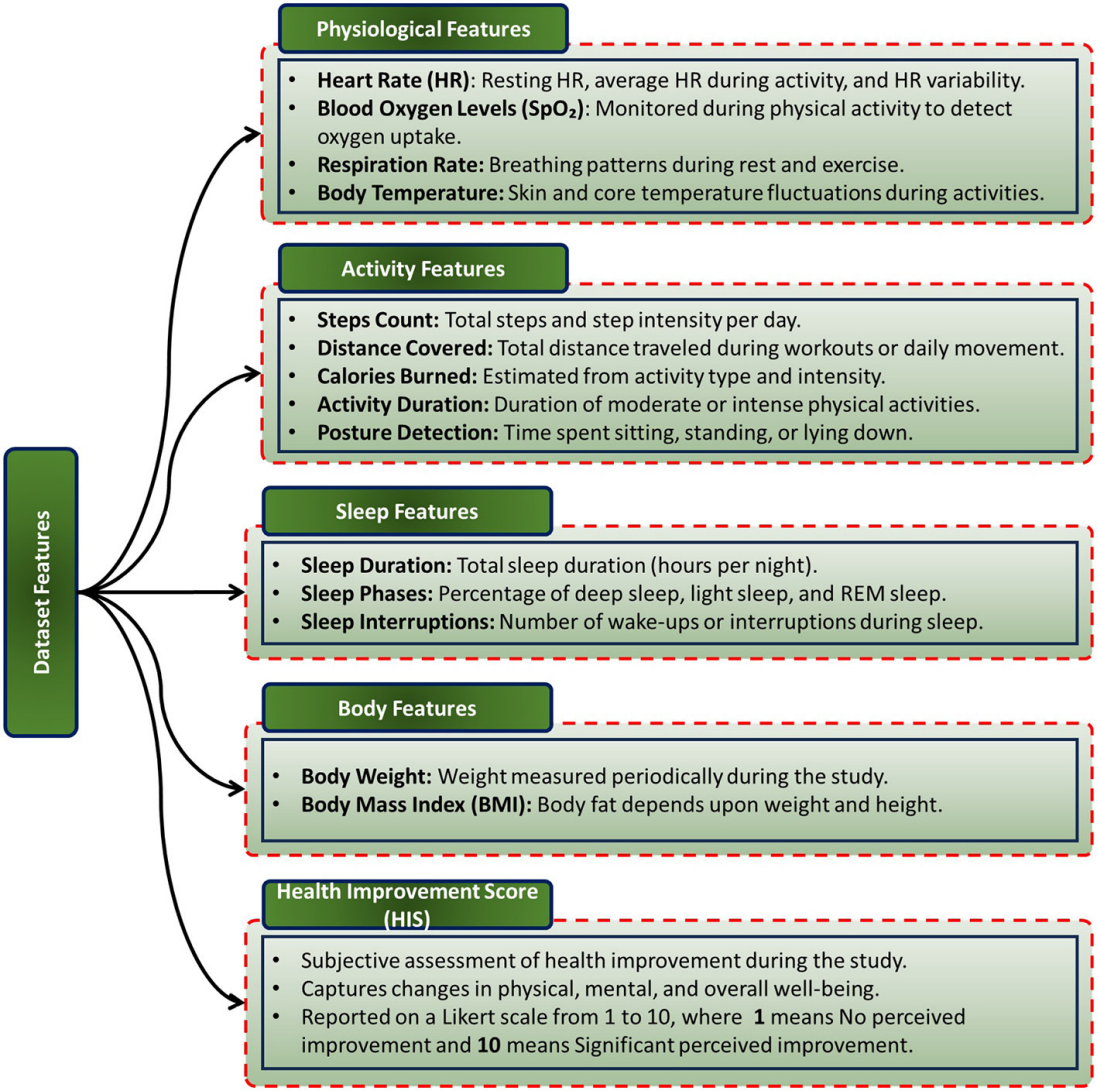
The dataset features, categorized into physiological, activity, sleep, and body metrics, along with the target variable, i.e., Health Improvement Score (HIS).

Figure 2 illustrates all features of the dataset, including the target variable, providing a comprehensive overview of the data used for analysis. Together, these features form a multidimensional dataset designed to evaluate the interplay between various health determinants and the resulting perceived health improvement.

#### 2.1.1 Physiological features

Physiological data were meticulously gathered to assess health and fitness indicators. Heart rate (HR) measurements include resting HR, average HR during activity, and HR variability, providing insights into cardiovascular fitness and autonomic nervous system regulation. Blood oxygen levels (SpO_2_) were monitored during physical activity to assess oxygen uptake efficiency. Respiration rate data captured breathing patterns during both rest and exercise, while body temperature measurements recorded fluctuations in skin and core temperatures during various activities.

#### 2.1.2 Activity features

Activity-related metrics aimed to capture participants' movement patterns and energy expenditure. These features include total steps and step intensity per day, total distance traveled during daily movement or workouts, and calories burned, estimated based on the type and intensity of activity. Additionally, activity duration captured the time spent in moderate or intense physical activities, while posture detection recorded time spent sitting, standing, or lying down.

#### 2.1.3 Sleep features

Sleep data were integral to understanding participants' recovery and overall well-being. Features include total sleep duration (hours per night) and the percentage distribution of sleep phases, such as deep sleep, light sleep, and REM sleep. Sleep interruptions, measured as the number of wake-ups or disturbances during sleep, provided further insight into sleep quality.

#### 2.1.4 Body features

Body-related metrics included body weight, measured periodically during the study, and body mass index (BMI), calculated based on participants' weight and height. These features offered additional context for evaluating physical health trends over the study period.

#### 2.1.5 Target variable: Health Improvement Score (HIS)

The Health Improvement Score (HIS), the target variable, serves as a subjective measure of overall well-being. Participants self-reported their perceived health improvement on a Likert scale from 1 to 10, where 1 indicates no perceived improvement and 10 represents significant perceived improvement. This score encapsulates changes in physical, mental, and overall health over the study duration.

### 2.2 Proposed HIS prediction model

To predict the HIS, a novel time-series-based deep learning model was designed. This model uses the temporal relationships in the dataset's physiological, activity, sleep, and body features collected over the study period. The following section provides a detailed description of the proposed model.

The HIS prediction model is a sequence-to-sequence time-series network specifically tailored for health-related temporal datasets. The proposed model incorporates several elements that distinguish it from traditional time-series predictive models. With five layers, the proposed deep LSTM model captures complex hierarchical temporal dependencies in the data. This enables better feature representation across different time scales. The integration of an attention mechanism enables the proposed model to dynamically focus on the most relevant time steps. This improves its ability to capture key temporal patterns in health data.

The proposed model is tailored to fuse heterogeneous data types (e.g., physiological, activity, sleep, and body features). It helps the proposed model to capture cross-domain interactions critical for health prediction tasks. Finally, a modified regularization technique, DropConnect (24), is applied to the recurrent layers. This enhances the generalization ability of the model without overfitting. The attention weights provide interpretable insights into the contribution of various features and time steps to the prediction. This aids in the understanding of health improvement trends.

Overall, the proposed architecture can be decomposed to following layers.

- **Input layer:** time-series data from all features, structured as **X** = {*x*_1_, *x*_2_, …, *x*_*T*_}, where *T* is the total number of time steps.
- **Recurrent layers:** five stacked Long Short-Term Memory (LSTM) (25, 26) layers are used to capture both short-term and long-term dependencies in the time-series data. The state of the LSTM is updated as:

(1) $$\begin{array}{l} h_{t}=\text{LSTM}\left(x_{t},h_{t-1},c_{t-1}\right), \end{array}$$

where *h*_*t*_ is the hidden state, *c*_*t*_ is the cell state, and *x*_*t*_ is the input at time step *t*.

- **Attention mechanism:** an attention mechanism (23) is integrated into the final LSTM output to dynamically weigh the importance of different time steps in predicting the HIS. The attention mechanism enables the model to automatically identify and focus on the most informative time steps, such as days with sudden spikes in heart rate or unusually poor sleep, by assigning them higher weights in the context computation. This dynamic weighting both boosts predictive accuracy and yields interpretable insights into which temporal events most influence each individual's HIS. The attention scores are computed as:

(2) $$\begin{array}{l} \alpha_{t}=\frac{\exp\left(h_{t}^{⊤}W_{a}h_{T}\right)}{\overset{T}{\underset{k=1}{\sum }}\exp\left(h_{k}^{⊤}W_{a}h_{T}\right)}, \end{array}$$

where α_*t*_ is the attention score for time step *t*, and *W*_*a*_ is the attention weight matrix. The final context vector is:

(3) $$\begin{array}{l} c=\overset{T}{\underset{t=1}{\sum }}\alpha_{t}h_{t}. \end{array}$$

- **Fully connected layers:** dense layers are applied to the context vector to learn feature interactions and produce a scalar output:

(4) $$\hat{y}=\sigma (Wc+b),$$

where *ŷ* is the predicted HIS, **W** is the weight matrix, *b* is the bias vector, and σ is the activation function.

#### 2.2.1 Loss function

The model uses a composite loss function that combines Mean Squared Error (MSE) with a temporal smoothness regularization term. The loss function is defined as:


(5) $$ℒ=\frac{1}{N}\sum_{i=1}^{N}{\left(y_{i}-{\hat{y}}_{i}\right)}^{2}+\lambda \frac{1}{T-1}\sum_{t=1}^{T-1}{\left({\hat{y}}_{t}-{\hat{y}}_{t+1}\right)}^{2},$$

where *N* is the number of samples. *y*_*i*_ is the true HIS value. ŷ_*i*_ is the predicted HIS value. λ is a regularization weight to balance the MSE and smoothness terms. *T* is the number of time steps in the prediction window.

The second term penalizes abrupt changes between consecutive predictions. This ensures smooth temporal outputs that better reflect realistic health improvement trends.

#### 2.2.2 Implementation details

The model was implemented using TensorFlow/Keras, a widely used deep learning framework, due to its flexibility and ease of use for designing complex neural network architectures. The implementation involved:

- **Data preprocessing:** time-series data were normalized to a range of [0, 1] to ensure faster convergence during training. Missing values were imputed using forward-fill methods (27), and temporal sequences were segmented into overlapping windows to capture meaningful trends.
- **Model training:** training was conducted on a high-performance NVIDIA A100 GPU with 40 GB of memory, ensuring faster computations and enabling the use of larger batch sizes. The Adam optimizer was employed for gradient-based optimization, offering an adaptive learning rate for stable training dynamics.
- **Regularization techniques:** to mitigate overfitting, Dropout and DropConnect techniques were applied during training. These methods randomly deactivated neurons and weights in the LSTM and dense layers, promoting robust learning.
- **Early stopping:** early stopping was implemented with a patience value of 10 epochs, monitoring validation loss to prevent overtraining and improve generalization.
- **Model checkpointing:** the model weights with the best validation loss were saved during training, ensuring optimal parameters were retained for final evaluation.

#### 2.2.3 Hyperparameter tuning

Hyperparameters were fine-tuned using a grid search approach (28) to identify the optimal configuration for the model. The grid search explored various combinations to select the most suitable parameters for maximizing validation performance. The tuned parameters and their respective ranges are shown in Table 1.

**Table 1:** Hyperparameter tuning results.

**Parameter**	**Range**	**Selected value**
Number of LSTM layers	3 to 6	5
Hidden units per layer	64 to 256	128
Learning rate	0.0001, 0.001, 0.01	0.001
Dropout rate	0.1 to 0.4 (increments of 0.1)	0.2
Batch size	32, 64, 128	64

To determine the ideal model depth, we conducted a grid search over 3-6 stacked LSTM layers (see Table 1). We found that 3- and 4-layer networks underfit the complex, multi-domain time-series–yielding higher validation errors, while a 6-layer network, although achieving very low training loss, showed signs of overfitting (a growing train-validation gap and longer convergence times). The 5-layer configuration provided the best trade-off: it minimized validation loss, converged efficiently, and kept computational cost manageable on our A100 GPU. Consequently, we adopted five LSTM layers for all reported experiments.

## 3 Performance analysis

The proposed and competitive models were implemented and trained in a Python environment utilizing TensorFlow (version 2.11). Training was conducted on a high-performance computing cluster equipped with an NVIDIA A100 Tensor Core GPU featuring 40 GB of memory, a 64-core AMD EPYC processor, and 256 GB of RAM, running on the Ubuntu 20.04. The hyperparameters of the competitive models were determined through a combination of trial-and-error experimentation and by referencing the configurations reported in their respective papers.

### 3.1 Comparative analysis

The performance of eight models, including ARIMA (16), SVR (18), ARIMA-SVR (19), SARIMA (17), LSTM (20), GRU (21), CNN-LSTM (22), and the Proposed Model, was evaluated on three key metrics. These metrics are Mean Absolute Error (MAE), Root Mean Squared Error (RMSE), and the Coefficient of Determination (*R*^2^). The results are visualized in the boxplots above, which highlight the strengths and weaknesses of each model.

**Figure 3:**
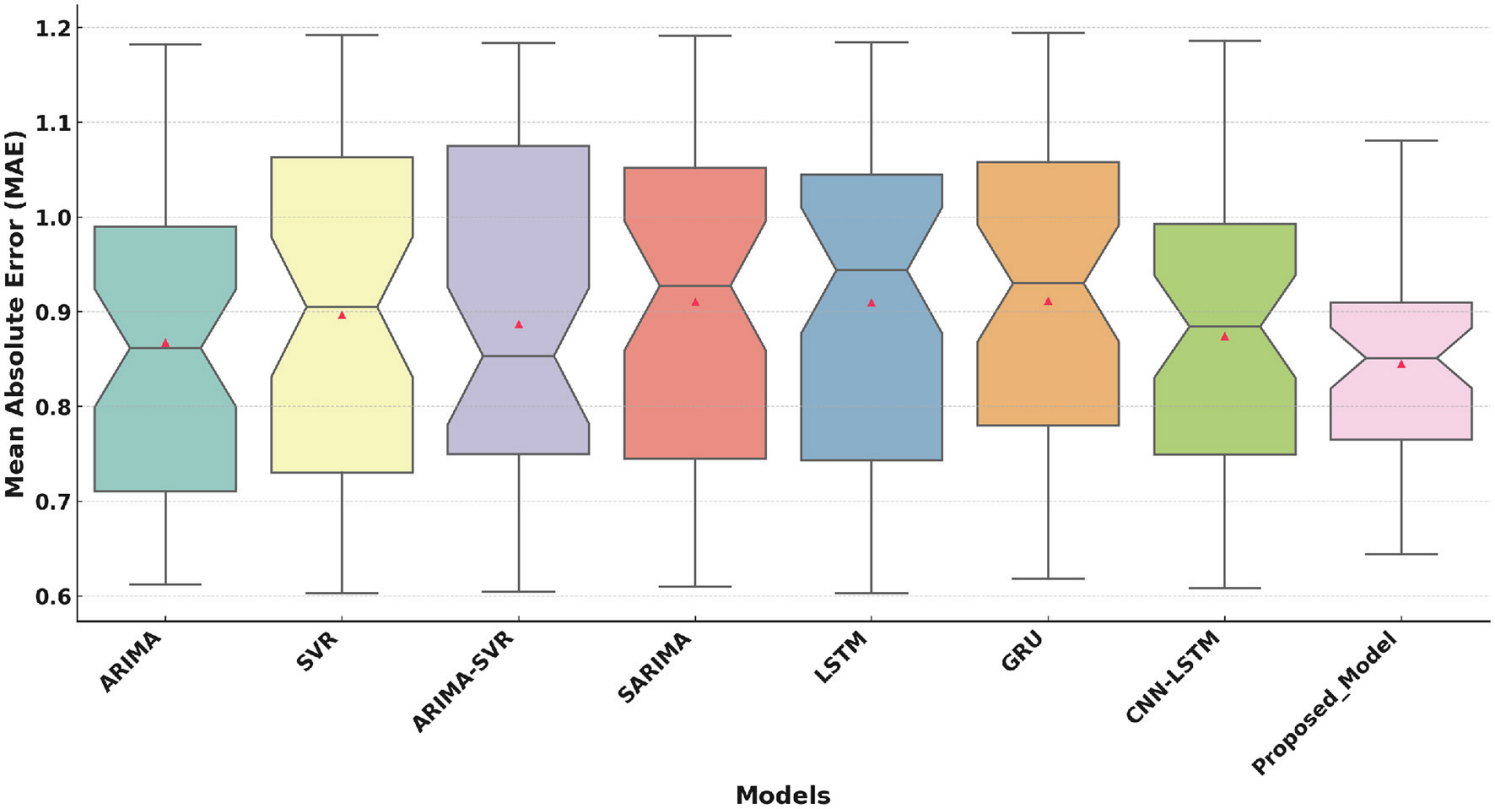
Mean Absolute Error (MAE) comparison of the Proposed Model with traditional and deep learning models.

Figure 3 shows that the Proposed Model achieves a median MAE of approximately 0.82. This is lower than all the competitive models. While some variability is present, the overall distribution of the proposed model is narrower. Traditional models such as ARIMA and SVR show higher medians with broader variability. Hybrid models like ARIMA-SVR and deep learning models (LSTM, GRU, CNN-LSTM) perform moderately well. However, they are consistently outperformed by the proposed model.

**Figure 4:**
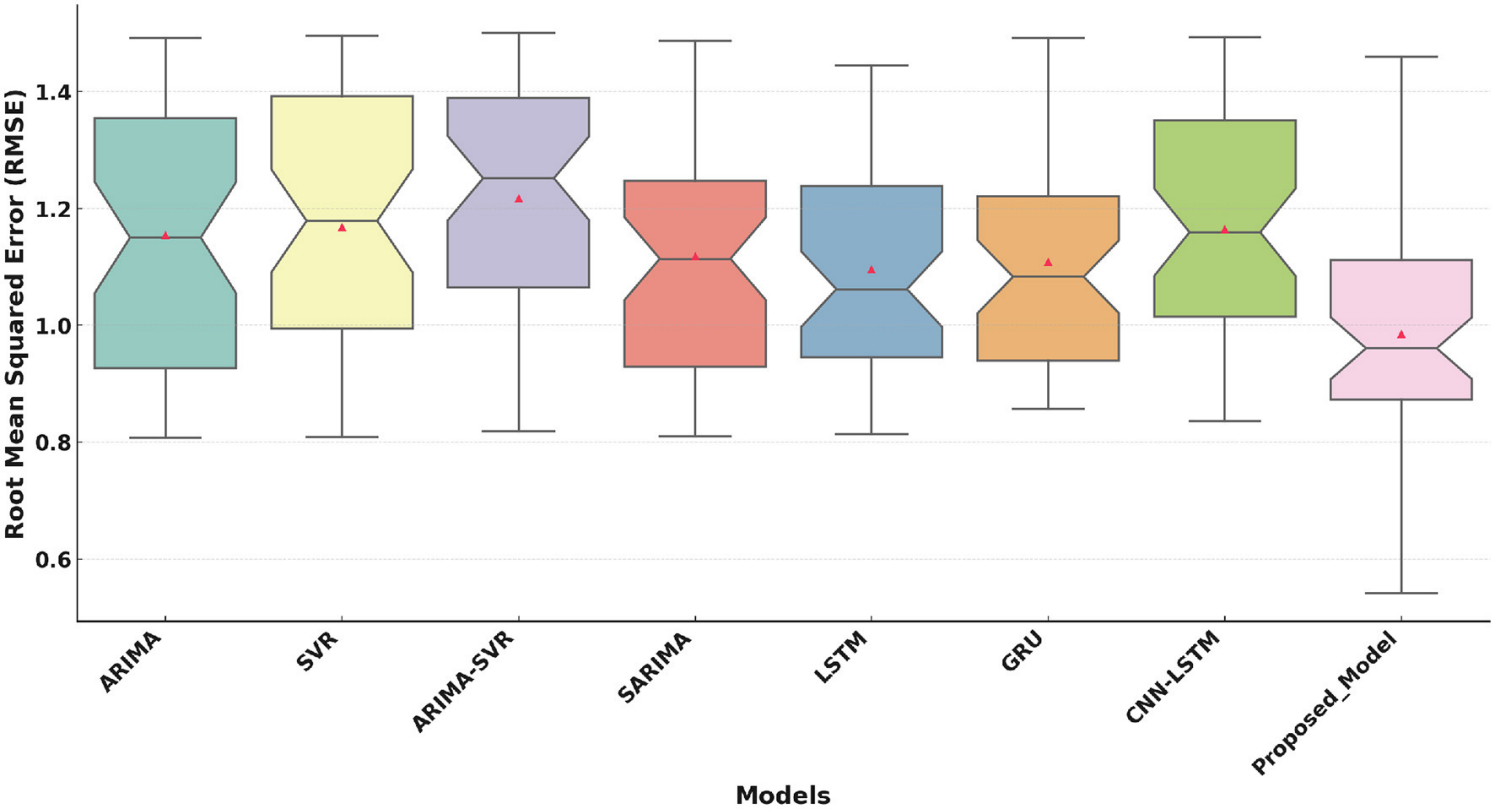
Root Mean Squared Error (RMSE) comparison of the Proposed Model with traditional and deep learning models.

Figure 4 highlights that the Proposed Model has a median value of approximately 0.98. This demonstrates its superior ability to minimize error. The performance of the proposed model shows slightly higher variability compared to MAE. However, its median RMSE remains significantly lower than traditional and deep learning models. This indicates its robustness and reliability across conditions.

**Figure 5:**
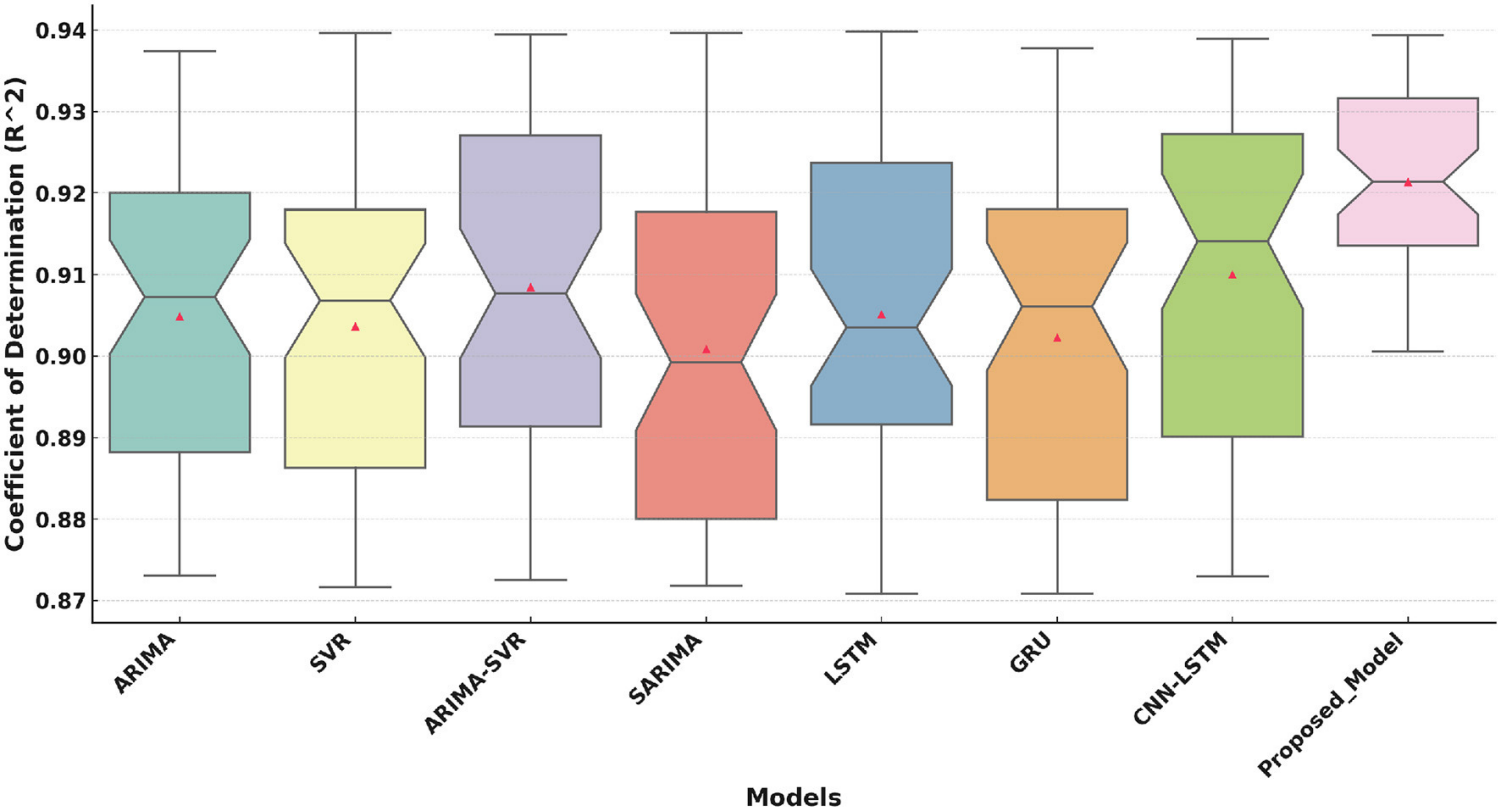
Coefficient of Determination (*R*^2^) comparison of the Proposed Model with traditional and deep learning models.

Figure 5 reveals that the Proposed Model achieves a high median value of 0.92 for *R*^2^. This metric reflects its strong ability to explain variance in the target variable. Although all models have *R*^2^ values in the range of 0.87 to 0.94, the proposed model consistently performs best. The narrow confidence intervals (notches) in the proposed model suggest its predictions are more reliable than those of the other models.

**Figure 6:**
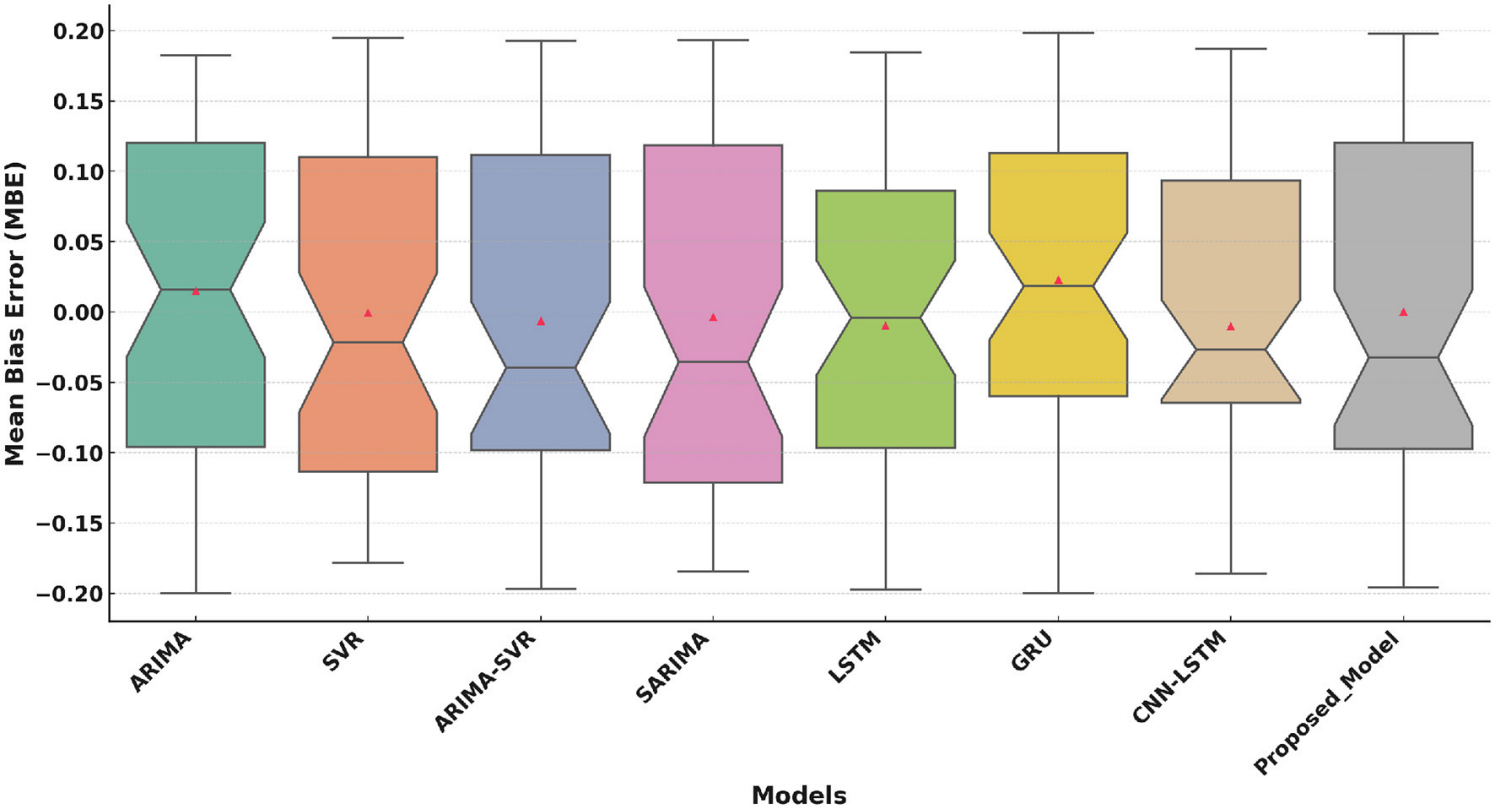
Mean Bias Error (MBE) comparison of the Proposed Model with traditional and deep learning models.

Figure 6 highlights the MBE performance. The Proposed Model exhibits a lower median bias (closer to 0) that reflects reduced systematic error compared to other models. Traditional models (ARIMA, SVR) and hybrid models (ARIMA-SVR) exhibit higher bias, while deep learning models show improved but still higher variability. The proposed model's narrow distribution indicates greater consistency and reliability.

**Figure 7:**
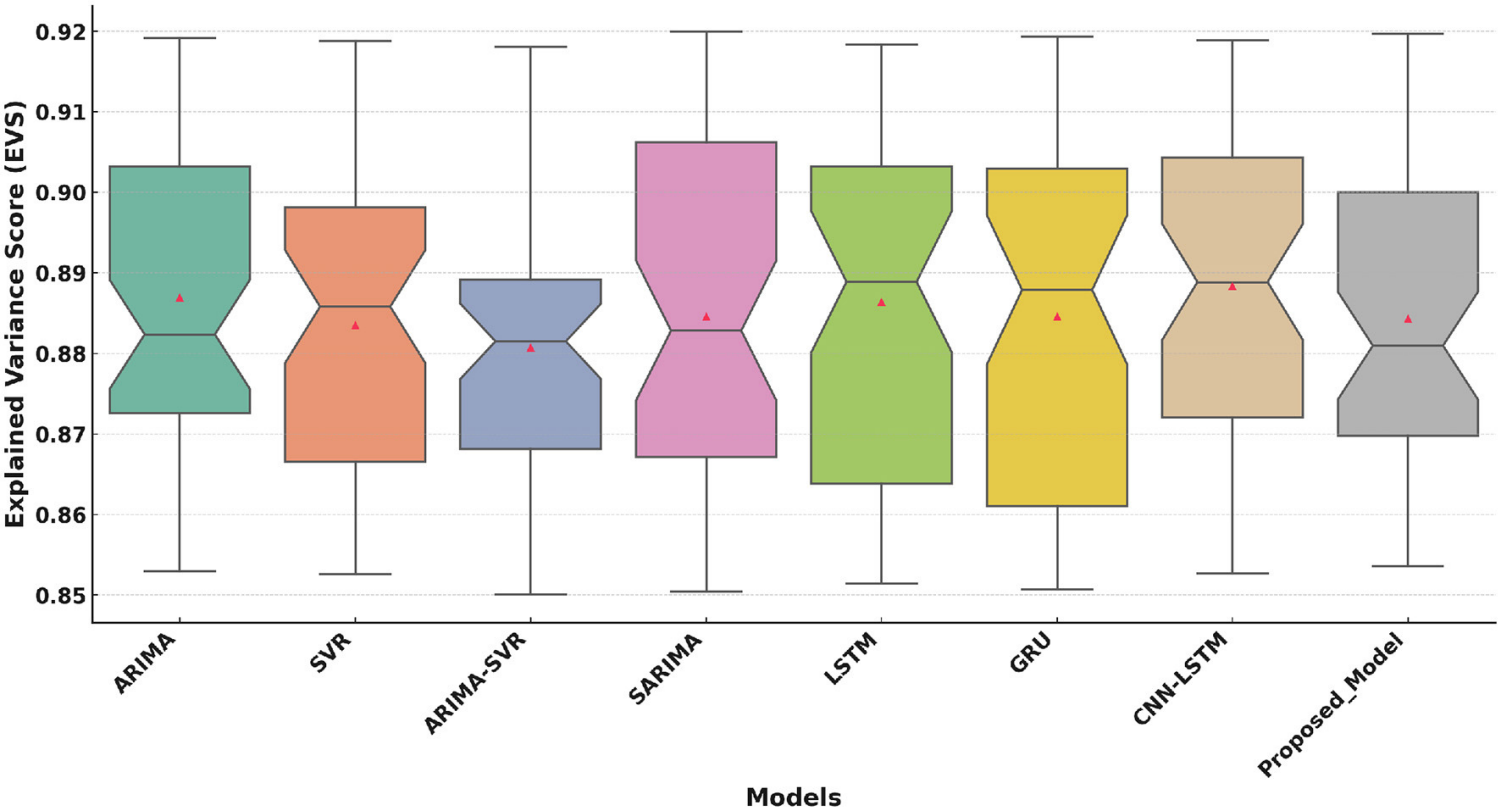
Explained Variance Score (EVS) comparison of the Proposed Model with traditional and deep learning models.

Figure 7 compares the EVS across models. The Proposed Model achieves a median EVS of approximately 0.95. This demonstrates the proposed model's ability to explain the variance in the prediction results. Traditional models (e.g., ARIMA, SVR) perform less effectively, with lower EVS values and greater variability. Deep learning models (e.g., LSTM, GRU, CNN-LSTM) show improved EVS scores but remain outperformed by the proposed model, whose narrow distribution signifies high reliability.

### 3.2 HIS score prediction

**Figure 8:**
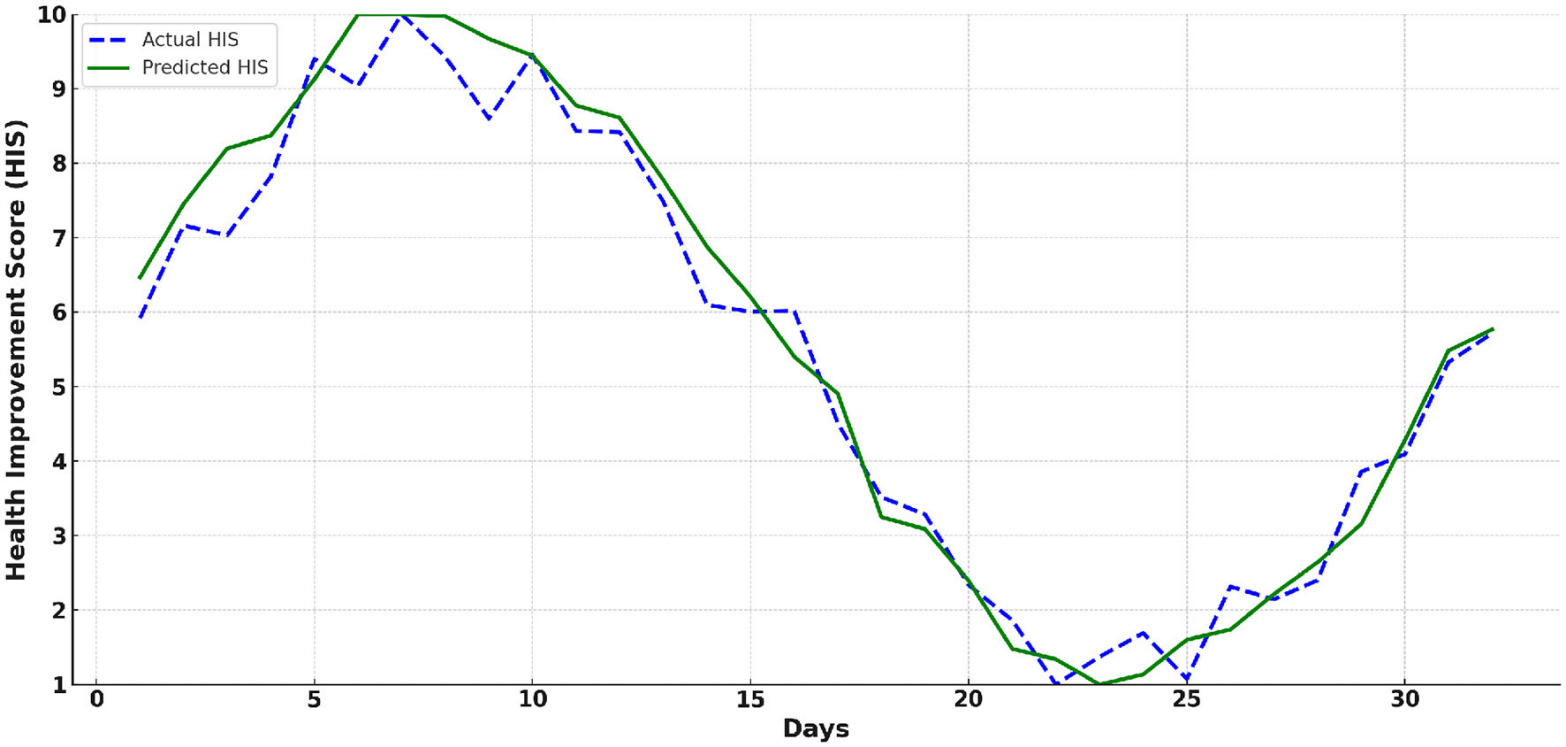
The trend of actual and predicted Health Improvement Score (HIS) values over a 32-day period.

Figure 8 illustrates the trend of actual and predicted HIS values over a 32-day period. It reflects the impact of sports and physical training on health improvement. The Proposed Model closely tracks the actual HIS values, with minor deviations observed, indicating high predictive accuracy. The figure also highlights the average benefits of sports and physical training on health. Initially, both types of training lead to noticeable improvements in HIS scores, followed by a slight decline. Subsequently, the HIS scores resume an upward trend, showcasing the sustained positive effects of consistent training.

**Figure 9:**
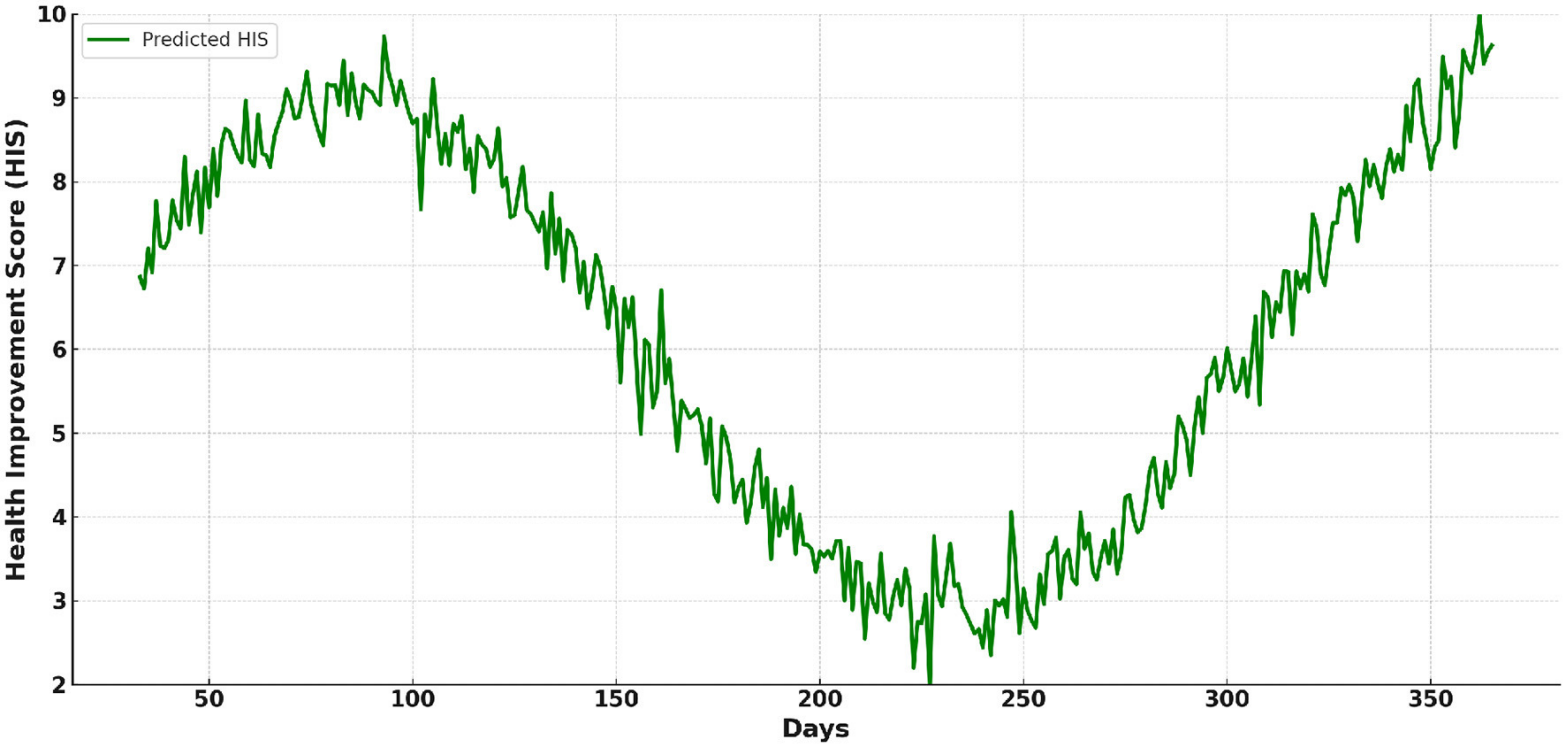
The predicted trend of Health Improvement Score (HIS) values starting from Day 33.

Figure 9 illustrates the predicted trend of HIS values starting from Day 33. It reflects the model's ability to project long-term health improvement trends. The predicted HIS values show a steady upward trajectory over time, indicating sustained health benefits from consistent sports and physical training interventions. It highlights the practical applicability of the model in monitoring and guiding health interventions.

The predicted HIS values display a generally upward trajectory over time, with occasional periods of slower growth or slight declines. This pattern reflects that as individuals continue regular sports and physical activity, the rate of improvement in HIS may not be as consistent as in the initial phases. This is because the body becomes healthier and adapts to the routine, leading to diminishing incremental gains.

The consistent upward and occasional downward trend in HIS reflects the natural adaptation of the body to regular sports and physical activities. As the body becomes healthier, the rate of improvement slows, leading to fluctuations in progress. This is a normal physiological response, where significant initial gains taper off as the body reaches a more optimized state of health.

## 4 Discussion and recommendations

### 4.1 Discussion

The findings of this study demonstrate the effectiveness of the proposed HIS prediction model in accurately forecasting health improvement trends based on sports and physical training data. The integration of deep learning with time-series analysis has enabled the model to capture complex temporal dependencies and interactions among physiological, activity, sleep, and body metrics. The results from performance evaluation show that the proposed model outperforms traditional statistical models such as ARIMA, as well as machine learning-based models like SVR and LSTM.

The boxplot analysis of key performance metrics, including MAE, RMSE, and *R*^2^, highlights the superiority of the proposed approach. The lower MAE and RMSE values indicate improved prediction accuracy, while the higher *R*^2^ value confirms the model's ability to explain variance in health improvement scores effectively. The results further reveal that deep learning-based approaches, particularly models utilizing attention mechanisms, provide a more reliable and adaptable solution for predicting HIS trends.

An important observation from the HIS trend analysis is the dynamic nature of health improvement. The initial phase of training yields noticeable gains, followed by periods of adaptation where the rate of improvement slows. This aligns with physiological principles where the body adapts to consistent training, leading to reduced marginal gains over time. These findings emphasize the need for periodically adjusting training programs to maintain steady progress in health improvement.

Additionally, the proposed model offers a practical application for health professionals, trainers, and policymakers. By using AI-driven insights, customized training programs can be developed, focusing on an individual's specific needs. This personalized approach ensures that interventions are both effective and sustainable.

### 4.2 Recommendations

Based on the findings of this study, several recommendations can be made to further improve health monitoring and personalized fitness interventions:

1. **Personalized training adjustments:** the model indicates diminishing returns in health improvement over time. Fitness trainers and health professionals should adapt training programs by incorporating variations in intensity, duration, and exercise type to maximize long-term benefits.
2. **Integration with wearable devices:** the proposed model can be further enhanced by integrating real-time data from wearable health monitoring devices. This would allow continuous tracking and real-time feedback, leading to more dynamic and responsive health interventions.
3. **Multi-population studies:** while the current dataset includes a diverse set of participants, future research should explore the effectiveness of the model across different age groups, fitness levels, and medical conditions. This would improve the generalizability of the model's predictions.
4. **AI-based health applications:** the proposed model can be deployed as a mobile or web-based application, enabling individuals to track their health improvement scores in real time. AI-based recommendations can further guide users in optimizing their fitness routines.

### 4.3 Limitations

Despite the promising results, there are several limitations to this study that should be acknowledged:

1. **Limited duration of study:** the dataset used in this study spans 32 days, which may not fully capture long-term health improvement trends. A longer study period would allow for more comprehensive analysis of sustained training effects.
2. **Dependence on self-reported HIS:** the target variable (i.e., HIS) is based on self-reported perceptions. This may introduce subjective bias. Future research should explore objective biomarkers to validate the HIS.
3. **Generalizability to different populations:** the study sample, while diverse, may not represent all demographic groups. Factors such as age, pre-existing health conditions, and genetic predispositions could influence health improvements differently.
4. **Computational complexity:** the deep learning architecture, particularly with attention mechanisms and multiple LSTM layers, requires high computational resources. This may limit real-time deployment on low-power devices such as smartphones or fitness wearables.

## 5 Conclusions and future outlook

This study proposes an HIS prediction model using LSTM networks with an attention mechanism to analyze temporal health trends influenced by sports and physical training. The proposed model integrates physiological, activity, sleep, and body metrics to provide accurate and interpretable predictions of health improvement. By incorporating LSTM networks and an attention mechanism, the model captures complex temporal dependencies and ensures a personalized and adaptive approach to health monitoring. The proposed model is compared against ARIMA, SARIMA, SVR, LSTM, GRU, and CNN-LSTM using MAE, RMSE, *R*^2^, and EVS. The experimental results demonstrate that HIS model achieves 22.8% lower MAE, 19.3% lower RMSE, 6.5% higher *R*^2^, and 7.9% higher EVS compared to competitive models. Furthermore, the HIS trend analysis reveals that consistent training leads to sustained health benefits, with initial rapid improvements followed by adaptation phases. This finding supports the need for dynamic and personalized fitness programs to maintain long-term health progress.

For future research, several key directions are suggested:

1. **Extended longitudinal studies:** future studies should involve extended monitoring periods (e.g., 6 months to 1 year) to analyze the long-term impact of sports and physical training on health improvement.
2. **Hybrid AI models:** the integration of reinforcement learning or hybrid deep learning models (e.g., Transformer-based architectures) can further enhance prediction accuracy and adaptability.
3. **Real-time implementation:** developing real-time prediction systems using cloud-based can make the model accessible to a broader audience.
4. **Multimodal data fusion:** incorporating additional health indicators such as diet, stress levels, and genetic predispositions can improve the robustness of the HIS prediction model.
5. **Ethical and privacy considerations:** as AI-based health applications grow, ensuring data privacy, ethical AI use, and regulatory compliance will be critical areas of focus.

## Data Availability

The original contributions presented in the study are included in the article/supplementary material, further inquiries can be directed to the corresponding author.

## References

[1] Yan J. Retracted: Evaluation method of public physical training quality based on global topology optimization deep learning model (Retracted Article). J Environm Public Health. (2022) 2022:4043876. 10.1155/2022/4043876 36159772 PMC9499795

[2] Xu D Song Y Meng Y Istvan B Gu Y. Relationship between firefighter physical fitness and special ability performance: predictive research based on machine learning algorithms. Int J Environ Res Public Health. (2020) 17:7689. 10.3390/ijerph17207689 33096792 PMC7589610

[3] Roliak AO. Professional education of teachers in physical training and health: the experience of Denmark. Pedag Phys Cult Sports. (2020) 24:143–50. 10.15561/26649837.2020.0307

[4] Qiao R Hu Y. Investigation and analysis on current sport life of regular college students in Jiangsu Province China. In:Zhang J Jiang Y Zou Y Chem J, editors. Proceedings of the 2010 International Symposium on Children and Youth Fitness and Health. Xi'an: Xi'an Physical Education University (2010). p. 404–407.

[5] Shingo N Takeo M. The educational experiments of school health promotion for the youth in Japan: analysis of the ‘sport test' over the past 34 years. Health Promot Int. (2002) 17:147–60. 10.1093/heapro/17.2.147 11986296

[6] Garkusha SV. Current trends in the health of children and young people in learning. environments. Pedagog Psychol Med-Biolo Prob Phys Training Sports. (2013) 10:7–11. 10.6084/m9.figshare.9759

[7] Zhaowei C. On cultivating college students sports interest in PE class. In:Zhao H Khan N, editors. International Symposium 2014 - *Common Development of Sports and Modern Society*. Beijing: Beijing Sport University Press (2014). p. 73–75.

[8] Eloirdi A Mammad K Arfaoui A Ahami A. The commitment: A determinant basic mental skill in student's performance in Physical Education and Sport. Pedagog Psychol Med-Biolo Prob Phys Training Sports. (2018) 22:246–51. 10.15561/18189172.2018.0504

[9] Hoffmann K Wiemeyer J Hardy S Goebel S. Personalized adaptive control of training load in exergames from a sport-scientific perspective towards an algorithm for individualized training. In:Gobel S Wiemeyer J, editors. Games for Training, Education, Health and Sports. Cham: Springer (2014). p. 129–140.

[10] Ford PR Yates I Williams AM. An analysis of practice activities and instructional behaviours used by youth soccer coaches during practice: exploring the link between science and application. J Sports Sci. (2010) 28:483–95. 10.1080/02640410903582750 20419591

[11] Khalajtsan AP. Laying the foundations of a culture of health as a pedagogical. Problem. Pedagog Psychol Med-Biolo Prob Phys Training Sports. (2014) 8:22–8.

[12] Bouquiaux O Thibaut A Beaudart C Dorban G Bertrand S Yildiz E. Dance training and performance in patients with Parkinson disease: effects on motor functions and patients' well-being. Sci Sports. (2022) 37:45–50. 10.1016/j.scispo.2021.03.004

[13] Kozina ZL Ilnitskaya AS Paschenko NA Koval MV. Integrated application of health improving methods of pilates and bodyflex for improving psychophysiological possibilities of students. Pedagog Psychol Med-Biolo Prob Phys Training Sports. (2014) 3:31–6. 10.6084/m9.figshare.936963

[14] Lukianova YS. School health approach to teaching and learning of students. Pedagog Psychol Med-Biolo Prob Phys Training Sports. (2015) 19:52–6. 10.15561/18189172.2015.0110

[15] Karapuzova ND. Health saving technologies in the training of future primary school. teachers. Pedagog Psychol Med-Biolo Prob Phys Training Sports. (2015) 19:39–45. 10.15561/18189172.2015.0108

[16] Shumway RH Stoffer DS. ARIMA models. In: Time Series Analysis and its Applications: With R Examples. Cham: Springer (2017). p. 75–163.

[17] Dubey AK Kumar A García-Díaz V Sharma AK Kanhaiya K. Study and analysis of SARIMA and LSTM in forecasting time series data. Sustain Energy Technol Assessm. (2021) 47:101474. 10.1016/j.seta.2021.101474

[18] Awad M Khanna R Awad M Khanna R. Support vector regression. In: Efficient Learning Machines: Theories, Concepts, and Applications for Engineers and System Designers. (2015). p. 67–80.

[19] Xu D Zhang Q Ding Y Huang H. Application of a hybrid ARIMA-SVR model based on the SPI for the forecast of drought–a case study in Henan Province, China. J Appl Meteorol Climatol. (2020) 59:1239–59. 10.1175/JAMC-D-19-0270.135865671

[20] Chang G Liu J. [Retracted] Analysis of factors related to adolescents' physical activity behavior based on multichannel LSTM model. Comput Intell Neurosci. (2022) 2022:1022421. 10.1155/2022/1022421 35832255 PMC9273359

[21] Dey R Salem FM. Gate-variants of gated recurrent unit (GRU) neural networks. In: 2017 IEEE 60th international midwest symposium on circuits and systems (MWSCAS). Boston, MA: IEEE (2017). p. 1597–1600.

[22] Ellouze A Kadri N Alaerjan A Ksantini M. Combined cnn-lstm deep learning algorithms for recognizing human physical activities in large and distributed manners: a recommendation system. Comput Mater Continua. (2024) 79:048061. 10.32604/cmc.2024.048061

[23] Niu Z Zhong G Yu H A. review on the attention mechanism of deep learning. Neurocomputing. (2021) 452:48–62. 10.1016/j.neucom.2021.03.091

[24] Wan L Zeiler M Zhang S Le Cun Y Fergus R. Regularization of neural networks using dropconnect. In: International Conference on Machine Learning. New York: PMLR (2013). p. 1058–1066.

[25] Al-Selwi SM Hassan MF Abdulkadir SJ Muneer A Sumiea EH Alqushaibi A. RNN-LSTM: from applications to modeling techniques and beyond–systematic review. J King Saud Univers-Comp Inform Sci. (2024) 36:102068. 10.1016/j.jksuci.2024.102068

[26] Wen X Li W. Time series prediction based on LSTM-attention-LSTM model. IEEE Access. (2023) 11:48322–31. 10.1109/ACCESS.2023.3276628

[27] Thakker ZL Buch SH. Effect of missing value handling pre-processing techniques onmachine learning algorithms to predict particulate matter concentration for Gandhinagar, Gujarat, India. Mach Intellig Res. (2024) 18:1110–22. 10.32628/IJSRST52411150

[28] Wojciuk M Swiderska-Chadaj Z Siwek K Gertych A. Improving classification accuracy of fine-tuned CNN models: impact of hyperparameter optimization. Heliyon. (2024) 10:e26586. 10.1016/j.heliyon.2024.e26586 38463880 PMC10920154

